# Genome sequencing of ovine isolates of *Mycobacterium avium *subspecies *paratuberculosis *offers insights into host association

**DOI:** 10.1186/1471-2164-13-89

**Published:** 2012-03-12

**Authors:** John P Bannantine, Chia-wei Wu, Chungyi Hsu, Shiguo Zhou, David C Schwartz, Darrell O Bayles, Michael L Paustian, David P Alt, Srinand Sreevatsan, Vivek Kapur, Adel M Talaat

**Affiliations:** 1 National Animal Disease Center, USDA-Agricultural Research Service, Ames, Iowa, USA; 2 The Laboratory of Bacterial Genomics, Department of Pathobiological Sciences, University of Wisconsin-Madison, Madison, Wisconsin, USA; 3 Laboratory for Molecular and Computational Genomics, Department of Chemistry and Laboratory of Genetics, UW Biotechnology Center, University of Wisconsin-Madison, Madison, Wisconsin, USA; 4 Department of Veterinary Population Medicine and Department of Veterinary and Biomedical Sciences, University of Minnesota, St. Paul, Minnesota, USA; 5 Department of Veterinary and Biomedical Sciences and Huck Institutes of the Life Sciences, Penn State University, University Park, Pennsylvania, USA; 6 Department of Food Hygiene, Cairo University, Cairo, Egypt

**Keywords:** *M. paratuberculosis*, Evolution, Johne's disease, Genome, Optical mapping

## Abstract

**Background:**

The genome of *Mycobacterium avium *subspecies *paratuberculosis *(*MAP*) is remarkably homogeneous among the genomes of bovine, human and wildlife isolates. However, previous work in our laboratories with the bovine K-10 strain has revealed substantial differences compared to sheep isolates. To systematically characterize all genomic differences that may be associated with the specific hosts, we sequenced the genomes of three U.S. sheep isolates and also obtained an optical map.

**Results:**

Our analysis of one of the isolates, *MAP *S397, revealed a genome 4.8 Mb in size with 4,700 open reading frames (ORFs). Comparative analysis of the *MAP *S397 isolate showed it acquired approximately 10 large sequence regions that are shared with the human *M. avium *subsp. *hominissuis *strain 104 and lost 2 large regions that are present in the bovine strain. In addition, optical mapping defined the presence of 7 large inversions between the bovine and ovine genomes (~ 2.36 Mb). Whole-genome sequencing of 2 additional sheep strains of *MAP *(JTC1074 and JTC7565) further confirmed genomic homogeneity of the sheep isolates despite the presence of polymorphisms on the nucleotide level.

**Conclusions:**

Comparative sequence analysis employed here provided a better understanding of the host association, evolution of members of the *M. avium *complex and could help in deciphering the phenotypic differences observed among sheep and cattle strains of *MAP*. A similar approach based on whole-genome sequencing combined with optical mapping could be employed to examine closely related pathogens. We propose an evolutionary scenario for *M. avium *complex strains based on these genome sequences.

## Background

*Mycobacterium avium *subspecies *paratuberculosis *(*MAP*) causes Johne's disease in sheep, cattle, goats and other ruminant animals. This disease is chronic in nature with multiple years separating the initial infection from clinical stages of disease [1]. The details of the pathogenic mechanisms occurring during this long incubation period still need further study, but it has been demonstrated that MAP colonizes the small intestine through invasion of both M cells and epithelial cells [2]. The disease is of considerable economic significance to livestock industries, particularly the dairy industry.

Generally, *MAP *is a genetically homogenous subspecies, especially among bovine, human and wildlife isolates [3-5]. However, three lineages of *MAP *have emerged following extensive molecular strain typing and comparative genomic studies-type I and type III strains (ovine) and type II (bovine) strains. The type III strains were originally called intermediate strains and are highly similar genetically, and thus, difficult to distinguish from type I strains. Early on, the type I (*MAP*-S) and type II (*MAP*-C) strains were distinguished based on their molecular fingerprints using IS*1311 *polymorphism [6], representational difference analysis [7], MLSSR typing [8-10] and *hsp65 *sequencing [11]. On the other hand, type III (a sub-lineage of the *MAP*-S strains) was genotyped based on *gyrA *and *gyrB *genes [12].

In addition to these recently published genotypic distinctions between "S" and "C" strains of *MAP*, phenotypic differences have been noted since the middle of the last century [4]. More recently, Motiwala et al. [13] have shown transcriptional changes in human macrophages infected with *MAP*-C, human and bison isolates induce an anti-inflammatory gene expression pattern, while the *MAP*-S isolates showed expression of pro-inflammatory cytokines. Furthermore, some of the ovine strains are pigmented [14]. The ovine and bovine strains likewise are distinct in their growth characteristics. The *MAP*-S strains are more fastidious and slower in their growth rate than the *MAP*-C counterpart. In contrast to *MAP*-C strains, the *MAP*-S strains do not grow readily on Herold's egg yolk media or Middlebrook 7H9 media that is not supplemented with egg yolk [15]. Nutrient limitation will kill *MAP*-S strains but it is only bacteriostatic for *MAP*-C strains [16]. On the transcriptional level, RNA extracted in low iron and heat stressed environments is divergent between *MAP*-S and *MAP*-C strains [17]. Recently, iron storage in low iron conditions was only observed in the *MAP*-C strains but not *MAP*-S strains [18]. Because of these well-documented phenotypic differences, we hypothesized that sequencing of the genomes of ovine isolates and comparing them to other genomes in the MAC group could provide some clues for these host-specific variations.

The *MAP*-C strain K-10 was sequenced in 2005 to obtain a complete genome 4.8 Mb in size [19]. It was subsequently found to possess an inversion due to misalignment that was resolved by optical mapping [20]. Very recently, draft sequences of ten *MAP *isolates have been reported with the presence of two large duplications, especially among human isolates [21]. Finally, another *M. avium *subspecies (strain 104) has also been sequenced but not published as yet. This genome of subspecies *hominissuis *is 5.4 Mb in size and greater than 95% homologous to the *MAP *K-10 genome [3,5,22]. Both of these genomes have served as reference genomes in the current project to assist in assembly, open reading frame (ORF) predictions, and annotation. With the help of next-generation sequencing and optical mapping, we were able to assemble a draft of the standard sheep strain of *MAP *S397 and compare its sequence to other clinical isolates from sheep or the K-10 strain. Interestingly, several inversion regions and single nucleotide polymorphisms distinguished the *MAP*-S strains from their *MAP*-C counterpart. Insights into the evolution of *MAP *strains have been gained through this analysis.

## Results

### Genome general features

Pyrosequencing indicated that the *MAP *strain S397 has a circular chromosome with at least 4,814,922 bp, a G + C content of 69.31% and contains 4,700 predicted open reading frames (ORFs). The majority of these genes (44.5%) were predicted [23] to encode cytoplasmic proteins (Additional file 1: Table S1) involved in various cellular functions and a minority of extracellular proteins (< 1%). The number of annotated genes in S397 was more than the bovine K-10 strain (Table 1) due to the different annotation methods used on each genome [19]. However, like *MAP *K-10, the S397 genome contains one rRNA operon and 46 tRNA genes representing all 20 amino acids. A detailed comparison between *MAP *strains K-10 and S397 as well as the human, *MAH *104 is shown in Table 1. The *de novo *assembly of the compiled S397 genome had an average sequencing depth of 24 × in 184 scaffolds (Additional file 2: Table S2). When aligned to the K-10 sequence, over 110 of these scaffolds are separated by a sequence gap of less than 500 bp suggesting the small size of most gaps. Furthermore, when gaps of 3.5 kb or less were ignored, we were able to assemble the whole genome into 3 scaffolds. The two largest sequence gaps are between contig00150c and contig00149c, which is estimated at 30.19 kb and the contig00082-contig00041c gap, which is estimated at 18.87 kb. Additional file 3: Table S3 gives an overview of the ordered scaffolds.

**Table 1:** A summary of the genomic features of *M. avium *subspecies isolates from different hosts

	MAP K-10	MAP S397	MAH 104
Origin	Bovine	Ovine	Human
Genome size (bp)	4,829,781	4,814,922	5,475,491
DNA scaffolds	1	184	1
G + C (%)	69.30	69.31	68.99
Protein coding (%)	91.56	91.31	88.66
Total genes	4,415	4,700	5,305
Total protein coding genes (PCG)	4,350	4,642	5,240
PCG without function prediction	3,014	1,085	1,732
PCG connected to KEGG pathways	1,215	1,249	1,303
tRNAs	46	46	46
rRNA operon	1	1	1

Analysis of the two additional genomes sequenced in this study (JTC1074 and JTC7565) revealed more than 99% identity to the S397 genome sequence (Table 2). A de novo assembly of these genomes sequenced using Illumina platform produced an average sequence depth of 60 ×. As expected, no significant differences were found between the common features of the 3 sequenced sheep isolate genomes. In fact, there were no gene differences; hence all three genomes were identically annotated. Similar to other sequenced mycobacterial genomes, *dnaA *was assigned the first locus tag (MAPs_00010).

**Table 2:** Reference genome assembly of clinical ovine isolates using simulated *MAP *S397 genome

	JTC1074	JTC7565
Reference organism	MAP S397	MAP S397
Reference length	4,766,015	4,766,015
Consensus length	4,753,502	4,742,737
%Homology^a ^to S397	99.74	99.51
%Homology to K-10	98.79	98.56
Average Coverage^b^	59.89	62.77
Standard deviation	32.36	34.65
Non-specific matches read count^c^	1387	1461
Paired read distance distribution	140-360	120-380
No. of SNPs	70	53

^a^Homology% was calculated as: consensus length divided by reference length and then multiplied by 100

^b^Average coverage is the average of all the reads in each area in the consensus sequence

^c^Non-specific match read counts are those reads that can be matched more than one place in the reference genome and such reads were randomly placed in one of the matched spots

The IS elements usually play a role in the genomic diversity among strains of mycobacteria [24] and could act as a good target for molecular diagnostics [25]. Similar to K-10, the S397 genome has all the well-studied insertion sequences (e.g. IS*900*, IS*1311 *and IS*_map02*). IS*900 *is generally considered a *MAP *specific element that was originally discovered in 1989 [26,27]. A total of 17 copies of IS*900 *were found in the S397 genome, which is identical to the K-10 strain. Another element, IS*_map02*, is a *MAP *specific insertion sequence that was discovered by sequencing the K-10 genome. A total of 6 copies of IS*_map02 *are present in both S397 and K-10. Likewise, IS*1311 *is present 7 times in each genome. No IS elements were found to be unique to one or the other genome.

### Organization of the *MAP *S397 genome

**Figure 1:**
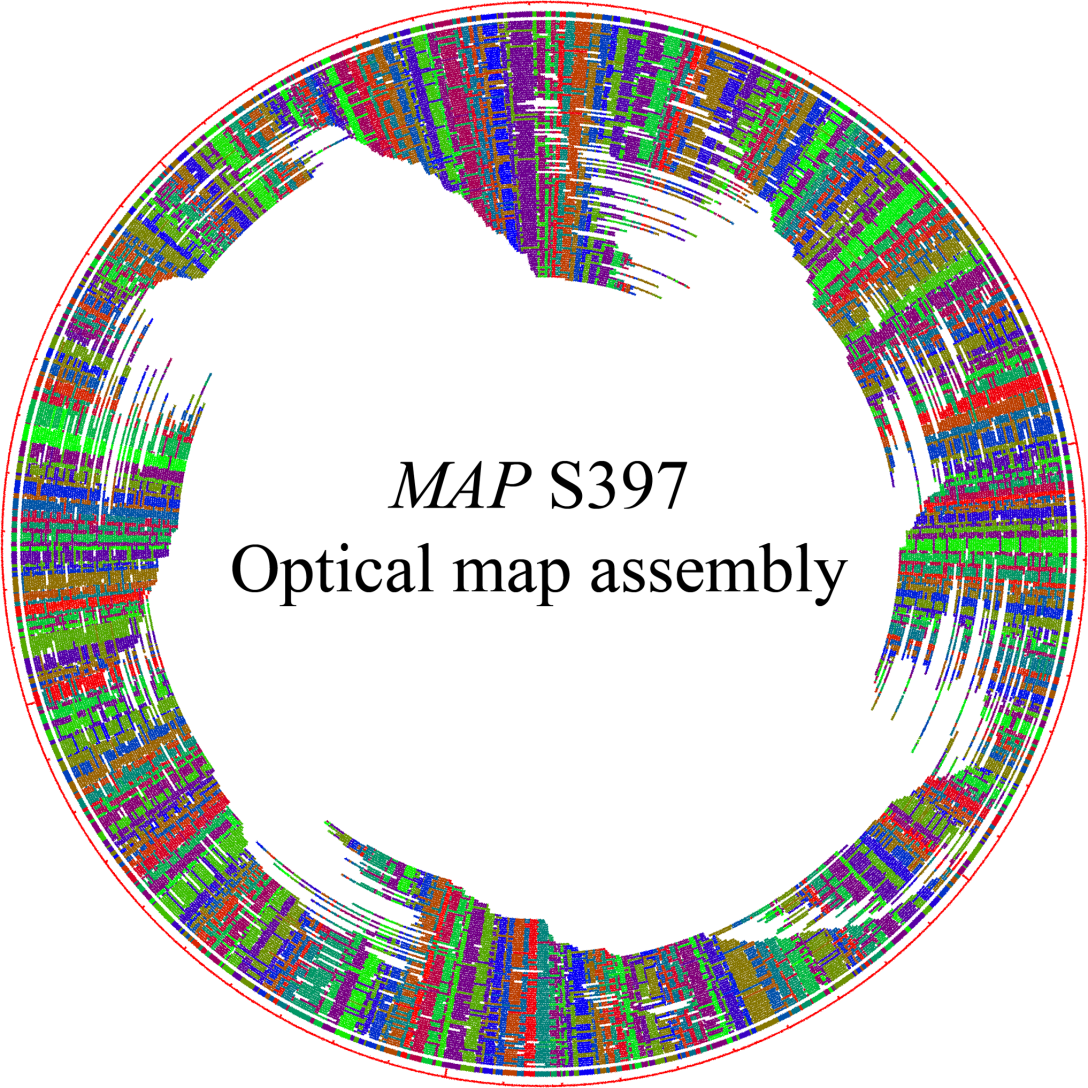
**Optical map of the *MAP *S397 genome**. A total of 905 optical contigs were assembled into one circular consensus map, which has a 58-fold genome coverage and totaled 4.95 Mb. Optical contigs are represented by arcs of various lengths. Each arc is intersected by radiating lines that represent *Bsi*WI cutting sites, and arbitrary colors represent homologous overlapping fragments.

Sequence analysis alone was not sufficient to decipher the synteny of the genome. Previously, we used an optical mapping protocol to confirm the organization of the *MAP *K-10 genome [20]. A similar strategy was used to analyze the genome of S397. The raw optical map dataset comprised 2,950 single molecule maps with a total mass of 784.5 Mb, and an average molecule size of 333.6 Kb (Figure 1). After assembly, the compiled optical map contained 905 single molecule optical maps (301.9 Mb; total mass), which covers the genome 58 ×. After a G + C content adjustment by a factor of 0.95, the estimated size of *MAP *S397 optical map is 4.95 Mb, which is slightly higher than the sequence data suggested. However, if the estimated sequence gaps are added in, the estimated sizes are very similar.

**Figure 2:**
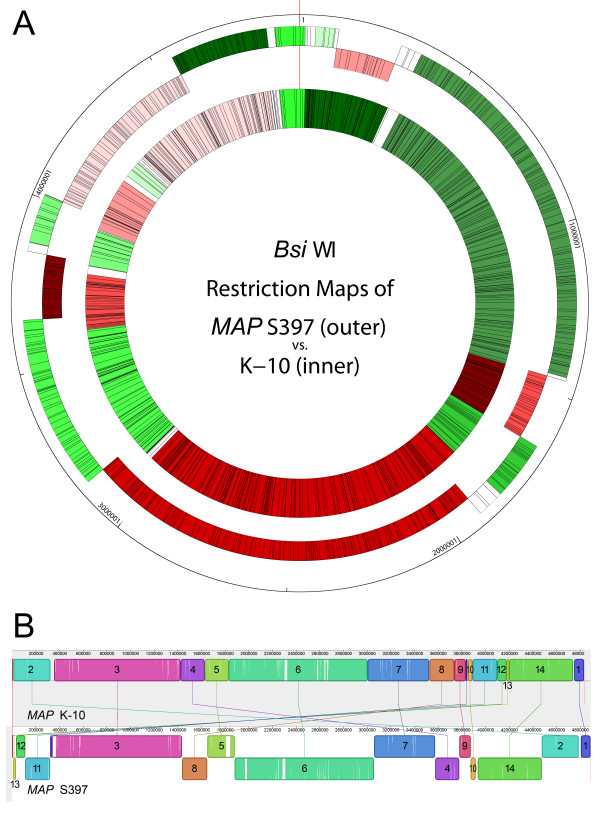
**Comparative genome analysis of K-10 and S397 *MAP *strains**. **(A) **Comparison of the *Bsi*WI restriction maps between K-10 (inner circle) and S397 (outer circle). Each box represents a restriction fragment. Green boxes are regions in the same direction and red boxes are regions that are inverted between the two genomes. White boxes are fragments that are not aligned. The red thin line at 12 o'clock is the locus of the gene *dnaA*. **(B) **Mauve alignment of all 184 scaffolds of S397 (bottom) with the complete genome of K-10 (top). The colored boxes represent homologous regions present in each genome, which are also connected by lines. Blocks below the centerline of the S397 genome indicate regions with inverse orientation. Regions outside the blocks lack homology between the genomes. Within each block there is a similarity profile of the DNA sequences and the white areas indicate sequences specific to a genome. The scale is in base pairs.

To our surprise, there were 7 inversions that are larger than 22 kb when the S397 genome was compared to the sequenced genome of K-10 compiled by Wynne [20,28]. The total size of these inversions spanned 2.4 Mb of the S397 genome. Individual sizes of those inversions range from 22 to 1,174 kb. As shown in Figure 2B, homologous segments between *MAP *K-10 and S397 are represented by color boxes and to each segment a number was assigned. Detail information of each segment is shown in Table 3. Thirteen out of the 14 segments have at least one IS element on the flanking regions (Figure 2).

**Table 3:** Boundaries and flanking ORFs of aligned segments between *MAP *K-10 and *MAP *S397 genomes

	**K-10 coordinates by Wynne **[**28**]		**K-10 coordinates by Li **[**19**]		Flanking ORF		Approx. size (Kb)	Alignment between K-10 and S397
ID	Left end	Right end	Left end	Right end	Left end	Right end		
1	4751793	11164	4748984	11164	IS*900*	IS_MAP03 and IS*1311*	92	Forward
2	13889	325818	4197078	3885146	IS_MAP03 and IS*1311*	No known IS element	312	Forward
3	357239	1424396	3853725	2786561	IS_MAP02	No known IS element	1067	Forward
4	1424397	1627687	2786560	2583268	No known IS element	ISmav2	203	Inverted
5	1629051	1828408	2581904	2382587	ISmav2	IS*1311*	199	Forward
6	1829822	3004096	2381173	1206891	IS*1311*	IS*1311*	1174	Inverted
7	3005512	3524059	1205475	686927	IS*1311*	ISmav2	519	Forward
8	3525433	3737882	685553	473103	ISmav2	No known IS element	212	Inverted
9	3737883	3833853	473102	377132	No known IS element	No known IS element	96	Forward
10	3849713	3890501	361272	320484	IS_MAP02	No known IS element	41	Inverted
11	3890502	4098807	320483	112177	No known IS element	IS*1311*	208	Inverted
12	4100223	4176993	110761	33991	IS*1311*	IS*1311*	77	Forward
13	4177670	4199835	33287	11158	IS*1311*	IS*1311*	22	Inverted
14	4201310	unknown^1^	4198493	unknown^1^	IS*1311*	N/A	562	Inverted

**Figure 3:**
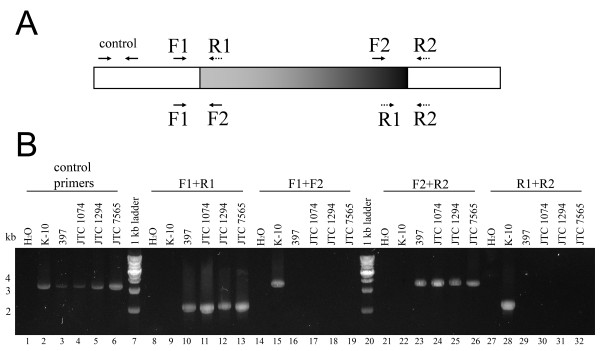
**PCR analysis of a 648-kb inverted region **[**20**]**between genomes of *MAP *bovine type strain (K-10), and four ovine strains (S397, JTC1074, 1294 and 7565)**. **(A) **A diagram showing the inverted region (gray-to-black gradient box) and location of primers used in the PCR analysis. All primers were designed according to the published *MAP *K-10 genome sequence [19]. **(B) **PCR results on an ethidium bromide stained agarose gel. Lanes loaded with PCR products amplified with original primer pairs F1 + R1 (lane 8-13) or F2 + R2 (lane 14-19) show no PCR products from the cattle strain (lane 9 and 22) but a 2.1-kb and 3.6-kb band from the sheep strains (lane 10-13 and 23-26), respectively. Lanes with products amplified with switched primer pairs F1 + F2 (lane 14-19) or R1 + R2 (lane 27-32) show a 3.6-kb and 2.3-kb fragment from the cattle strain (lane 15 and 28) but no product from the sheep strains (lane 16-19 and 29-32), respectively. The opposite PCR amplification pattern between the cattle and sheep strains confirmed that this segment is inverted between these 2 genomes.

Similar to our analysis of inversions discovered in the K-10 strain, we used a PCR-based approach to examine two of the inversion breakpoints in the S397 genome (Figure 3), which are the right end of segment ID #1 and the left end of segment ID#2 (Table 3). As expected, our PCR analysis confirmed the inversion predicted in the genome of K-10 and S397 strains. Because these inversions were readily identified from the optical map and sequence alignment data, we did not attempt to confirm all of the inverted fragments by PCR. Despite these inversions, there is strong synteny between these genomes, underscoring their close relatedness. Both genomes share a number of large-scale clusters of homology where gene order is highly conserved (Additional file 4: Table S4).

### Genomic insertions

Further comparative sequence analysis identified several regions that are present in *MAP *S397 and *MAH *104, but not in *MAP *K-10 (Additional file 5: Table S5). The largest of these is a 9-kb gene cluster encompassing 13 ORFs (MAPs_15940-MAPs_16060). This region was partially identified by representation difference analysis and termed PIG-RDA20 for pigmented strain representational difference analysis-20, as detailed before [7]. It was also mapped to the *MAH *104 genome by Dohmann and coworkers [7] and was subsequently described by Semret and coworkers as large sequence polymorphism (LSP), LSP^A^4-II [29]. This region contains a copy of the IS*1311 *insertion sequence and within the *MAH *104 genome is flanked by an additional copy of IS*1311*. Another previously described LSP included 9 ORFs (MAPs_46190-MAPs_46270) and totals 6.6 kb. This region was partially identified as the PIG-RDA10 sequence and was mapped to a 16 kb segment of the *MAH *104 genome [7]. The full sequence was later identified as LSP^A^18 [29], which is equivalent to MAV island 24 [3]. An interesting feature of LSP^A^18 is that it begins and ends with a transcriptional regulator. Eight other LSPs containing 4 or more ORFs not present in K-10 were also observed (Table 4). Overall, a total of 70 ORFs were present in *MAP *S397 but absent in the *MAP *K-10 genome (Additional file 5: Table S5).

**Table 4:** Large sequences present in the three sheep strain genomes but absent in *MAP *K-10.

LSP		Location				Gene Content
**Name**	**Synonym**	**Start**	**Stop**	**Size**	**Contig**	**ORFs**

LSP^s^1	LSP^A^4-II (RD20)	34,308	43,318	9.01	00036c	MAPs_15940-16060
LSP^s^2	LSP^A^18 (RD10)	116,723	123,369	6.64	00127c	MAPs_46190-46270
LSP^s^3		15,581	19,356	3.78	00035c	MAPs_14620-14660
LSP^s^4		124,533	128,162	3.63	00127c	MAPs_46290-46320
LSP^s^5		87,461	90,928	3.47	00126c	MAPs_17580-17610
LSP^s^6		4,736	7,731	2.99	00161	MAPs_40470-40500
LSP^s^7		2	2,889	2.88	00133c	MAPs_17640-17670
LSP^s^8		47,323	49,712	2.39	00199c	MAPs_02730-02760
LSP^s^9		70,659	72,500	1.84	00033	MAPs_23120-23150
LSP^s^10		5,879	7,457	1.58	00100	MAPs_42460-42490

*LSP *Large sequence polymorphism as identified before [29]

Size is in kilobases

Several new or only partially described LSPs common to *MAP *S397 and *MAH *104 strains were also identified. A good example here is the novel LSP found in *MAP *sheep and *MAH *104 genomes is comprised of 14 ORFs (MAPs_17580 - MAPs_17710), predicted to encode proteins involved in the biosynthesis of glycopeptidolipids [30]. This region in *MAP *S397 revealed the presence of four additional ORFs (*hyp, hlpA, dhgA *and *mtfC*) with homology to glycopeptidolipid biosynthesis genes immediately downstream. The additional 4 ORFs were also not present in the *MAH *104 sequence. Finally, a putative transcriptional regular labeled as MAPs_44910 is present in *MAP *S397. The protein encoded by this ORF has homology to the GntR-family of transcriptional regulators, which are widely distributed across bacterial species and regulate a variety of cellular processes [31,32].

### Genomic deletions

A second subset of sequence polymorphism was represented by 32 ORFs that were present in the *MAP *K-10 genome but absent from the genome of *MAP *S397 (Additional file 6: Figure S1). Several of these deletions have already been described earlier. The deletion encompassing MAP1485c-MAP1491 was previously identified by Marsh and coworkers as S strain deletion #1 in an Australian *MAP *sheep isolate [33] and by Semret and coworkers as LSP^A^20 [29]. An additional larger deletion in the *MAP *S397 included the cluster of ORFs between MAP1728c and MAP1744. This deletion was partially identified by Marsh and coworkers as RDA3 [34], and later fully described as S deletion #2 [33].

A novel deletion comprising the ORFs MAP1432-MAP1438c (partial) was identified in the current study as absent from *MAP *S397. This deletion, termed sΔ-1, was originally discovered by comparative genomic analysis and subsequently confirmed by PCR analysis. This gene cluster is predicted to encode four energy metabolism enzymes as well as a lipase (MAP1438c). MAP1432 encodes a hypothetical protein with homology to the REP13E12, a family of repetitive elements that were originally described in *M. tuberculosis *and have been shown to be targets of phage integration [35]. There is a homolog to MAP1434 that is present in S397 (MAPs_13210). The region around MAPs_13210 is not near the end of a contig and is nearly identical to an inverted stretch in K-10, thus leading to the conclusion that MAPs_13210 is only a homolog of MAP1434, but that the gene itself is not present in the S397 genome.

Interestingly, MAP2656 was initially identified as absent via microarray analysis [5] but sequencing of *MAP *S397 identified a homologue with 100% identity (MAPs_10401 & MAPs_10402). Likewise, MAP2325 was identified as being absent from Australian sheep isolates of *MAP *[33]. This ORF was not identified as missing from *MAP *S397 as sequencing confirmed the presence of an ORF (MAPs_34380) with 100% identity to MAP2325. These discrepancies may represent a geographic difference between *MAP *isolates recovered from sheep in Australia and the United States or it may be an error from the microarray experiment. These were the only observed differences between the microarray and sequence data. Overall, genomic alignments indicated the presence of a significant number of insertions and deletions between ovine and bovine strains of *MAP *that are suggested to be associated with their respective host.

### Evolutionary analysis of the *MAP *S397 genome

**Figure 4:**
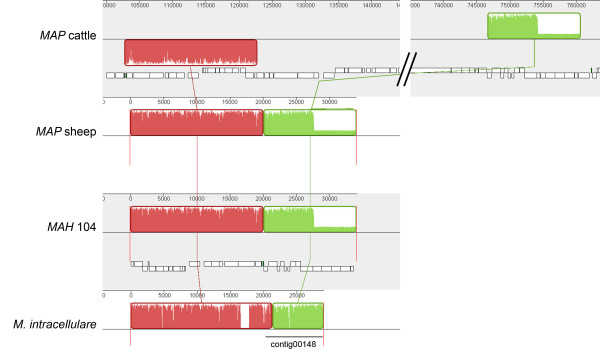
**Genomic alignment of inversion breakpoints among members of the *M. avium *complex**. Regions spanning the right breakpoint are depicted. The junction between the red and green boxes shown in the *MAP *sheep panel represents the breakpoint. Note that the breakpoint is within contig00148 of *M. intracellulare*. The alignment shows that the genome synteny among *MAP *sheep (S397), *MAH *104 and *M. intracellulare *is conserved.

Genomic insertions and deletions have been previously used to determine evolutionary relationships among MAC strains [36]. With the genome sequence of these ovine isolates of *MAP*, we can now add comprehensive SNP and inversion data to strengthen evolutionary hypotheses. Earlier genotyping of the *MAP S397 *utilizing SNP of *recF, gyrA *and *gyrB *genes indicated that this strain belong to the *MAP *type III, a sublineage of the *MAP-S *cluster of isolates [37]. To examine the evolutionary history of *MAP*, we analyzed the genome sequence of S397 compared to other clinical isolates circulating in sheep as well as the standard cattle strain, K-10. Our first level of analysis included the alignment of the S397 genome to that of the JTC1074 and JTC7565. This alignment resulted in identical genome organization of all three ovine isolates, as expected. Additionally, we examined the relationship among S397 (ovine origin) with both K-10 (bovine origin) and *MAH *104 (human origin). Such analysis identified several events of inversions and potential insertions/deletions between genomes belonging to the ovine isolates and other isolates of bovine and human origins (Figure 4). The optical map of S397 confirmed these inversions as well. Moreover, when the draft genome sequence of *M. intracellulare *was added to the comparison, the whole contig00148 (accession number GenBank: ABIN01000141) aligns to the region spanning the right breakpoint (Figure 4) of *MAH *104 and *MAP *ovine strains, an indication of a conserved genome synteny among *M. intracellulare, MAH *and *MAP *sheep strains, but distinct from *MAP *bovine strains.

**Figure 5:**
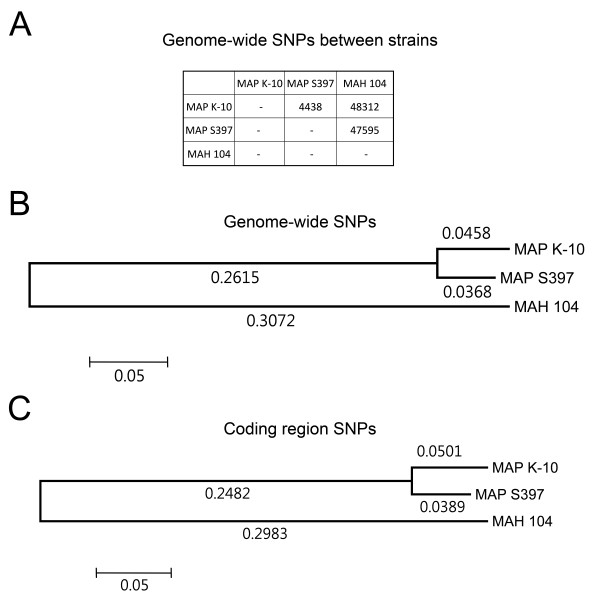
**Polymorphisms among *M. avium *complex (MAC) members**. **(A) **Table of the total single nucleotide polymorphisms (SNPs) present in each genome. **(B) **Phylogenetic relationship among MAC strains using all SNPs or those restricted to coding regions **(C)**. The trees were constructed using the Neighbor-Joining method [38]. Each tree is drawn to scale, with branch lengths (indicated below the branches) in the same units as those of the evolutionary distances used to infer the phylogenetic tree. The evolutionary distances were computed using the LogDet (Tamura-Kumar) method [39] and are in the units of the number of base substitutions per site. There were a total of 50,924 SNPs in the dataset for (B) and 38,546 SNPs in (C). Evolutionary analyses were conducted in MEGA5 [40].

In the second level of analysis on the nucleotide level, a core of 42 single nucleotide polymorphisms (SNPs) were present in both JTC isolates compared to S397. In addition, a very small number of unique SNPs in JTC1074 (N = 22) and JTC7565 (N = 11) were not present in any other genome in this study. Collectively, this small level of polymorphism indicates the clonal nature of ovine isolates, which contrasts sharply with the 4,438 SNPs between the ovine S397 and the bovine K-10 strains (Figure 5A). Additionally, when analyzing genome-wide SNPs, it appears that *MAP *S397 and K-10 split off recently from the *hominissuis *progenitor strain (Figure 5B). A similar result is obtained when SNPs are restricted to coding sequences (Figure 5C).

## Discussion

Comparative genomic hybridizations using DNA microarrays have revealed large sequence polymorphisms (LSPs) between *MAP-S *and *MAP-C *strains [36,41]. Two large deletions of an Australian sheep isolate were found by genomic hybridization to the *MAP *K-10 array [33]. One deletion encompassed 8 ORFs (MAP1485c-MAP1491) and a second deletion encompassed 17 ORFs extending from MAP1728c to MAP1744. These deletions relative to the bovine strains were later observed in U.S. ovine *MAP *isolates [5,13]. Construction of a *MAP *array containing *MAH *sequences revealed LSPs in the ovine strains that were missing in the bovine K-10 strain [5,42]. These documented differences formed the basis for whole-genome sequencing of a sheep isolate to enable comprehensive description of all genetic differences from *MAP-S *and *MAP-C *strains. We took advantage of next-generation sequencing technology combined with optical mapping [20] to decipher the complete genome of *MAP *isolates from sheep flocks raised in the USA. Our analysis confirmed earlier polymorphisms among *MAP*-S and *MAP*-C strains and revealed novel regions of difference. Surprisingly, both genome sequencing and optical mapping showed remarkable differences between *MAP*-S and *MAP*-C strains despite the overall similarity in the clinical signs of Johne's disease in sheep and cattle. Recently, a study using a large number of *MAP *isolates provided an example of such a genomic polymorphism including 2 large regions of duplication, termed vGI-17 (containing 63 ORFs) and vGI-18 (containing 109 ORFs), observed in most *MAP*-C strains but not *MAP*-S isolates [21]. Both of these duplications were also missing in our sequenced *MAP*-S genomes as determined by PCR amplification using outward facing primers reported by Wynne et al. (data not shown).

There are 70 genes present in all three ovine isolates that are absent from the K-10 strain, an indication for *MAP *adaptation to specific hosts (in this case sheep). Analysis of additional ovine and bovine isolates is needed to strengthen any linkage between these genes with host association. Within this subset, we identified a surprising number of genes annotated as hypothetical proteins (N = 30). Six transcriptional regulators were also present among these genes with the remaining genes showing weak homology to sequences in the GenBank database. We hypothesize that these genes could be responsible for the observed phenotypic differences between ovine and bovine strains and warrant future studies to address this hypothesis.

**Figure 6:**
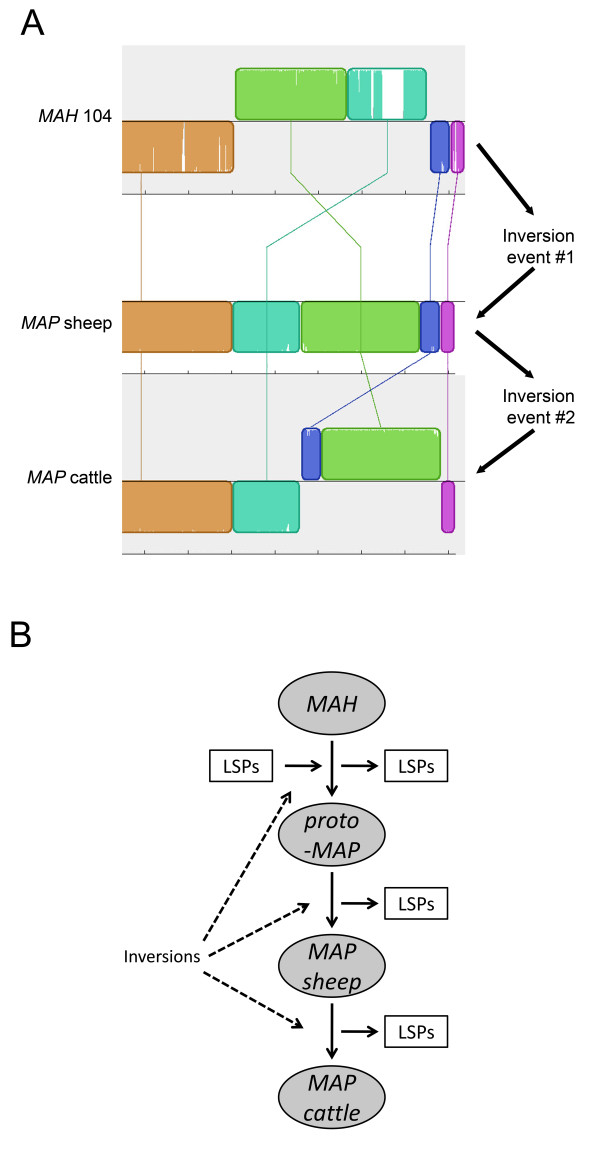
**An evolutionary scenario for members of the *M. avium *complex**. **(A) **Depicted is a two-step inversion process as one possible scenario explaining how *MAH *evolved into *MAP *K-10 through *MAP *S397. To examine evolutionary relationship among the MAC, genome alignment around the inversion segment is depicted with Mauve version 2.3.1 [44]. Divergence between *MAH *104 and *MAP *sheep strains or between *MAP *sheep strains and cattle strains would take only one inversion, whereas divergence between *MAH *104 and *MAP *cattle strains would need two independent inversion events. **(B) **Our proposed model for evolution of the *M. avium *complex.

Based on extensive genomic rearrangements between *MAP *bovine and ovine strains, we were able to provide a possible evolutionary scenario for members of the MAC group. A genomic region spanning the inversion of *MAP *bovine strains, *MAP *ovine strains and *MAH *104 are shown in Figure 4. To diverge into these three subspecies, the common ancestor appears to have undergone two independent genomic inversion events (Figure 6A). Specifically, it would take one inversion event to diverge between *MAH *104 and *MAP *sheep strains followed by a second inversion event between *MAP *sheep strains and the *MAP *cattle strain (Figure 6A). Therefore, assuming that one strain diverges into another strain by taking the shortest evolutionary path, it would be least likely that *MAH *directly evolved from *MAP *cattle strains or vice versa. This strongly suggests that *MAP *sheep strains are the intermediate taxon of the three. Data from Behr and coworkers suggest *MAH *104 is the ancestor strain [36]. Moreover, when the genome of *M. intracellulare *is added to the comparison, the genome synteny was conserved among *M. intracellulare, MAH *and *MAP *sheep strains, but not in *MAP *cattle strains. Thus, it is possible that the common ancestor of the MAC must resemble either *MAH *104 or *M. intracellulare*, and *MAP *bovine strains are the latest diverged strains among them with *MAP *S397 as an intermediary strain (Figure 6B). This model partially agrees with a hypothesis that suggests *MAH *differentiated into two lineages, *MAP *ovine and bovine strains, by delineating chronological genomic insertion/deletion events without considering other genomic rearrangement events [36]. Of the 70 genes in S397 that are absent in K-10, 57 are present in *MAH *104 and only 13 are absent from *MAH *104. Further genotyping of the S397 clustered this isolate with the group of *MAP*-S type III [37], a sub-lineage of the sheep strains. However, we prefer to maintain the *MAP*-S designation since the type III genotype was based on 3 SNPs present in a subgroup of sheep isolates with no distinctive clinical or pathological features. Finally, a recent study analyzing the sequence polymorphisms of IS*1311 *among the MAC also supports the hypothesis that *MAP *ovine strains are the intermediary taxa between *MAH *and *MAP *bovine strains [43].

## Conclusions

Genome sequencing of *MAP-S *strains have revealed extensive genome inversions and previously characterized deletions when compared to the K-10 strain. Furthermore, there appears to be a high degree of homology within US *MAP-S *strains as suggested by the remarkably low number of SNPs present in the three isolates sequenced. Evolutionary analysis based on whole genome sequencing suggests MAH is the progenitor strain, followed by MAP-S, followed by MAP-C strains.

Overall, Next-generation sequencing combined with optical mapping provided us with a high resolution tool to decipher the evolution of important pathogenic mycobacteria. Comparative sequence analysis of the *MAP *isolates from sheep has improved our understanding of the evolutionary history of members of MAC and provided the foundation for novel insights into the pathogenesis of this important pathogen. Similar approaches can be used to examine other closely related pathogens.

## Methods

### *MAP *ovine isolates

Isolates were cultured in Middlebrook 7H9 broth (BD Biosciences, San Jose, CA) media supplemented with 10% OADC (2% glucose, 5% bovine serum albumin factor V, and 0.85% NaCl), 0.05% Tween 80 and 2 μg/ml of Mycobactin J at 37°C [45]. The *MAP *ovine S397 strain was obtained from a Suffolk breed in Iowa. It was isolated from the distal ileum at necropsy in 2004. The other 2 sheep isolates of *MAP *(JTC1074 and JTC7565) were isolated from the intestine of infected sheep in Texas and obtained from the Johne's Testing Center at the University of Wisconsin-Madison. All isolates were genotyped using the IS*1311 *restriction endonuclease, which yielded the 2-band pattern typical of ovine strains [6].

### Genome sequencing

Genomic DNA was extracted as described in detail previously [3,46]. For the S397 strain, the DNA (1-5 μg) was sequenced using Roche 454 pyrosequencing (GS20 and FLX) at the National Animal Disease Center. A whole-genomic shotgun sequencing library was prepared according to Roche protocols. The library was used with the appropriate emulsion based PCR kits to produce sufficient beads for sequencing using the Roche Standard Chemistry GS-LR 70 sequencing kit. For the JTC1074 and JTC7565, the purified genomic DNA (~5 μg) of each strain was sent to Genomic Resource Center at the University of Maryland for Illumina whole genome sequencing (Multiplexing Sample Preparation oligonucleotide Kit) as outline before [47]. The adapters and indexing oligonucleotides were purchased from Illumina (5 Paired End Cluster Generation Kits-v4). The CLC Genomic Workbench software (version 4.0.3) was used to perform reference and de novo assembly on all sequenced genomes.

### Genome annotation

The S397 sequence was annotated using the Integrated Microbial Genomes Expert Review (IMG-ER) pipeline [48]. The sequences of the JTC isolates were annotated based on S397. Genes were each designated by the locus tag "MAPs" to distinguish it as a *MAP *sheep strain gene. This locus tag is followed by a five digit unique identifier, which incrementally increases by ten (i.e. MAPs_45660... MAPs_45670... MAPs_45680...). With this numbering configuration, additional genes can easily be added as they are discovered or when remaining gaps are closed.

### Genome comparison

The genome data for *MAP *K-10 (accession no. GenBank: NC_002944.2) and *M. avium *subsp. *hominissuis *(*MAH*) strain 104 (GenBank: NC_008595.1) were used in alignments in the Artemis and Artemis Comparison Tool (ACT) programs or Mauve 2.3.1 [49]. BLASTP analysis was used for similarity searches and protein sequence analysis. In addition, Mauve algorithm was used to align two or more genomes [50]. For detecting single nucleotide polymorphisms (SNPs) among sheep isolates, the CLC Genomic Workbench was used. The coverage range setting for each strain was at 10-55 reads, and the frequency of the mutation was at least in 50% of the reads.

### Optical mapping

Shotgun optical mapping, as previously described [20,51-55], was used to construct a physical restriction map for the S397 genome. Genomic DNA, in agarose inserts [56], was electroeluted into a solution containing a lambda DNA sizing standard (30 pg/μl), and then were mounted on cleaned, derivitized glass surfaces using a microfluidic device [57] followed by polymerization of a thin layer of polyacrylamide (3.3% containing 0.02% Triton X-100). Mounted DNA was digested with 20 units of *Bsi*WI (NEB, Ipswich, MA) for 1 to 2 hrs at 37°C. Fluorochrome-stained DNA fragments were imaged by fluorescence microscopy with a 63 × objective lens (Carl Zeiss, Thornwood, NY) and a high-resolution digital camera (Princeton Instruments, Trenton, NJ). Images were acquired and processed using "ChannelCollect" and "Pathfinder" -custom software [57] that converts captured images into map data sets. Bayesian inference and an efficient dynamic programming algorithm were also being used to fine-tune the parameters including standard deviation, digestion rate, false cut, and false match probability etc. [54,58,59]. The final circular optical map contig was built using an iterative assembly process [60] including rounds of pair-wise alignment (single molecule maps vs. seed maps; provisional assemblies) and assembly [52,54]. Due to the high G + C content of *MAP*, which skews fragment sizing by integrated fluorescence intensity measurement, the final maps were globally scaled (0.95) to correct this problem [20,61]. A laboratory software implementation of an optical map alignment algorithm [62] was used to align between optical fragments generated from *MAP *S397 and the *in silico *restriction maps of *MAP *K-10, which provided a whole-genome rearrangement comparison between the two genomes. This restriction framework was used to generate a temporary rearranged genome as the reference sequence to guide the assembly of *MAP *S397 *de novo *contigs with the function "move contigs" in Mauve 2.3.1 [49].

### PCR amplification of inversion breakpoints and deletions

PCR reactions were performed in 25-μl reaction mixture containing 1 M betaine (Sigma-Aldrich, St. Louis, MO), 50 mM potassium glutamate (Sigma-Aldrich), 10 mM Tris-HCl pH 8.8, 0.1% Triton X-100, 2 mM magnesium chloride, 0.2 mM dNTPs, 0.5 μM each primer, 0.5 U of GoTaq^® ^Flexi DNA Polymerase (Promega, Madison, WI) and 25 ng of genomic DNA. The amplification thermocycle started with an initial step of 94°C for 5 minutes followed by 5 cycles of 94°C for 30 s, 62°C for 30 s with 1°C decrease for each cycle and 72°C for 3.5 min, and followed by 30 cycles of 94°C for 30 s, 57°C for 30 s and 72°C for 3.5 min. PCR primers used for examining the breakpoints included control F: AAGCATCACCTGCATGAGC, control R: CGGGAATTTATCCGTTTCAG, F1: GGGATCGATCTTGACCACAT, R1: GTGCCTGGACTCGATTTTGT, F2: AAGAGGTCGGAGGTTCGAGT and R2: CGGTGAGAGATTTCGTCACA. Primers used to demonstrate the S397 sΔ-1 deletion included F18: CGTCTTCCCCGTCGTCGTTC, B24: CGATGAGAGTCCGTGCGTGG, F15: CGGCGGGCGGTCAGGGTTTG, B17: GCAGGTTGGGGTTCGGCTTG, F7: GGTGGTCGGCGTCCTCGTAG, B9: CGTCGTCACAGCGAAAACGG, F3: CCACCCGCCTCACACCACTC, B4: AGGACGCCGACCACCAAACG. Conditions for the amplifications are essentially as described immediately above except that Advantage GC Genomic LA PCR Polymerase kit (Clontech) was used for each reaction.

### Nucleotide sequence accession number

This Whole Genome Shotgun project has been deposited at DDBJ/EMBL/GenBank under the accession AFIF00000000.

## Competing interests

The authors declare that they have no competing interests.

## Authors' contributions

JPB, MLP and DPA sequenced the S397 genome. JPB and DOB annotated the S397 genome. JPB and CW integrated and analyzed data from the S397 contigs and optical mapping, performed genomic comparison, PCR confirmation as well as SNP, inversion and evolutionary analyses. CH, CW and AMT sequenced and analyzed genomes of isolates JTC1074 and JTC7565. SZ and DCS collected, assembled and aligned S397 optical map. JPB, CW and AMT wrote the manuscript. JPB, SS, VK and AMT assisted with the analysis. All authors read and approved the final manuscript.

## Supplementary Material

Additional file 1 **Table S1**. PSORT analysis of MAP S397 genes to determine their localization. Click here for file

Additional file 2 **Table S2**. A list of 184 genomic DNA scaffolds of *M*AP S397 genome. Click here for file

Additional file 3 **Table S3**. Synteny of annotated genes between *MAP *K-10 and *MAP *S397 genomes. Click here for file

Additional file 4 **Table S4**. *MAP *S397 genes that are absent in *MAP *K-10. Click here for file

Additional file 5 **Table S5**. *MAP *K-10 genes that are absent in *MAP *S397. All supplemental tables are in Excel format. Click here for file

Additional file 6 **Figure S1**. An overview alignment of *MAP *S397 scaffold assembly and *Bsi*WI restriction fragments. Click here for file
