# Supplementary material for: Genome sequencing of ovine isolates of Mycobacterium avium subspecies paratuberculosis offers insights into host association
Source: BMC Genomics. 2012 Mar 12;13:89. doi: 10.1186/1471-2164-13-89 (PMC3337245; doi:10.1186/1471-2164-13-89)
Supplement: Additional file 6 — Figure S1. An overview alignment of MAP S397 scaffold assembly and BsiWI restriction fragments. [file 1471-2164-13-89-S6.PDF]

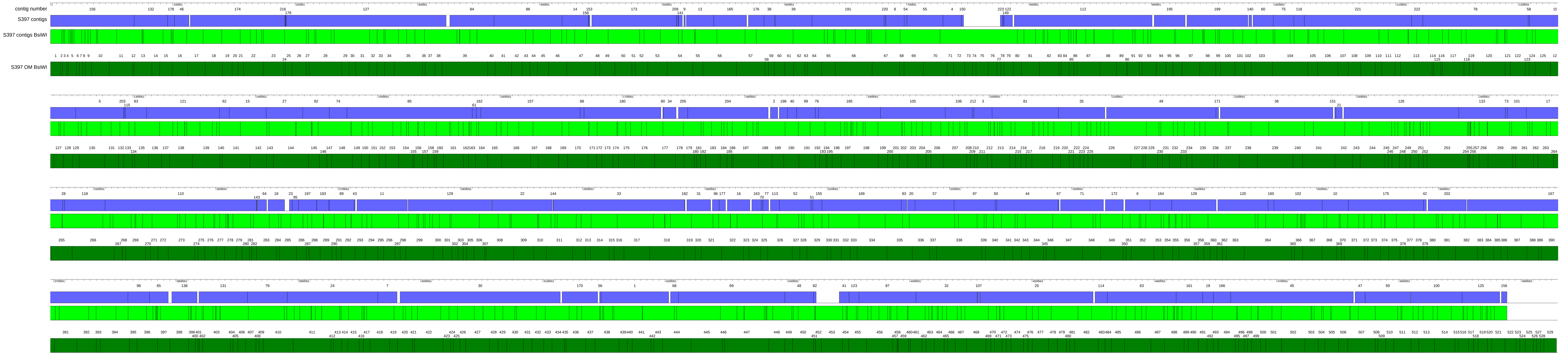
contig number
S397 contigs
S397 contigs BsiWI
S397 OM BsiWI
